# Supplementary material for: Accurate imputation of human leukocyte antigens with CookHLA
Source: Nat Commun. 2021 Feb 24;12:1264. doi: 10.1038/s41467-021-21541-5 (PMC7904773; doi:10.1038/s41467-021-21541-5)

Supplementary Information

Accurate Imputation of Human Leukocyte Antigens with CookHLA

Supplementary Table 1. Accuracy comparison of different versions of CookHLA. We imputed HapMap CEU (N=88) using the T1DGC (N=5,225) as reference. The version with the best accuracy is in bold font.

		CookHLA versions					
		Equiv. to SNP2HLA	CookHLA-vanilla	CookHLA-embed	CookHLA-HapMap	CookHLA-adapt	Full CookHLA
	Upgraded Hidden Markov model	X	O	O	O	O	O
	Genetic map	Not applicable	No map	No map	HapMap map	Adaptive map	Adaptive map
	Local embedding of markers	X	X	O	X	X	O
HLA-A		92.05%	97.16%	97.16%	96.59%	**98.86%**	**98.86%**
HLA-C		93.75%	**98.30%**	**98.30%**	**98.30%**	97.73%	**98.30%**
HLA-B		92.05%	**97.16%**	**97.16%**	**97.16%**	**97.16%**	**97.16%**
HLA-DRB1		90.34%	93.18%	93.75%	93.75%	94.32%	**94.89%**
HLA-DQA1		97.73%	98.86%	**99.43%**	**99.43%**	**99.43%**	**99.43%**
HLA-DQB1		94.89%	**97.16%**	**97.16%**	**97.16%**	**97.16%**	**97.16%**
Average		93.47%	96.97%	97.16%	97.06%	97.44%	**97.63%**

Supplementary Table 2. Pairwise accuracy benchmark using 10 reference panels. We collected 10 different reference panels of various populations and sizes, and tested each pair by assigning one as a reference and another as a target. The imputation accuracy was averaged

over all available HLA genes for each pair. In each cell, the first number is the accuracy of the full version of CookHLA, while the number in the parentheses is the accuracy of CookHLA-vanilla (CookHLA with only imputation engine upgrade, no genetic map is used).

		Reference panel									
		European			East Asian				South Asian	African	Ad Mixed American
		T1DGC	58BC	1000G EUR	Chinese	Korean	1000G EAS	Pan-Asian	1000G SAS	1000G AFR	1000G AMR
Target sample	T1DGC		91.3 (90.8)	93.3 (89.8)	80.4 (71.2)	75.1 (65.4)	73.0 (59.6)	78.7 (71.6)	85.1 (73.2)	88.7 (78.9)	91.0 (83.4)
	58BC	97.5 (97.4)		96.8 (94.4)	89.1 (85.1)	81.5 (72.7)	75.6 (63.0)	74.1 (69.1)	80.6 (78.0)	92.0 (80.2)	94.9 (88.2)
	1000G EUR	98.3 (97.5)	95.8 (94.4)		89.4 (85.6)	81.0 (76.6)	82.8 (72.0)	76.0 (71.1)	85.3 (78.7)	91.1 (86.2)	95.6 (88.1)
	Chinese	80.8 (79.0)	69.0 (63.4)	73.2 (65.1)		90.5 (82.2)	92.3 (83.5)	89.2 (83.5)	80.3 (71.6)	58.4 (51.1)	68.4 (62.0)
	Korean	90.0 (87.1)	71.9 (67.7)	74.0 (68.6)	95.4 (94.7)		95.2 (83.6)	90.6 (83.6)	77.9 (66.3)	61.9 (54.6)	71.3 (64.9)
	1000G EAS	91.3 (80.6)	61.2 (51.7)	68.8 (62.6)	94.4 (94.1)	90.8 (82.5)		Sample overlap	78.9 (74.8)	57.9 (50.5)	63.8 (60.5)
	Pan-Asian	90.0 (89.8)	61.0 (60.0)	64.3 (61.8)	87.5 (88.9)	78.4 (75.6)	Sample overlap		83.8 (74.6)	58.1 (51.0)	59.9 (54.4)
	1000G SAS	94.0 (90.5)	78.2 (74.0)	82.9 (73.4)	88.5 (82.3)	79.5 (73.8)	87.0 (81.8)	88.7 (81.8)		74.9 (62.8)	79.4 (68.2)
	1000G AFR	89.3 (84.4)	68.9 (62.5)	74.8 (67.8)	59.1 (57.5)	45.0 (39.8)	50.2 (42.8)	47.6 (38.3)	53.3 (45.0)		85.9 (67.7)
	1000G AMR	89.3 (88.4)	78.9 (78.2)	82.7 (77.5)	77.0 (76.0)	67.0 (61.4)	68.7 (62.4)	64.8 (58.7)	72.4 (64.7)	80.0 (73.7)	

Supplementary Table 3. Computation time of different methods. We used 2.1Ghz CPU cores with 8Gb memory per core. The computation time for obtaining adaptive genetic map using MACH is described in parenthesis for CookHLA.

	SNP2HLA	CookHLA (Beagle v4.1)		CookHLA (Beagle v5.1)		HIBAG-prefit	
Custom reference adaptability	O	O		O		X	
CPU core usage (# thread)	1	1	9	1	9	1	7
Memory assigned	8Gb	8Gb	72Gb	8Gb	72Gb	8Gb	56Gb
T1DGC ref (N=5,225) / 1000G EUR (N=503) (# Overlap SNP = 5,539)	2.0 hours	19.4 hours (+ 42mins)	2.6 hours (+ 42mins)	1.4 hours (+ 42mins)	10 mins (+ 42mins)	34 mins	10 mins
Chinese ref (N=9,773) / Korean target (N=413) (# Overlap SNP = 3,595)	33.2 hours	29.1 hours (+ 31mins)	4.5 hours (+ 31mins)	1.4 hours (+ 31mins)	11 mins (+ 31mins)	11 mins	3 mins

Supplementary Figure 1. Imputation accuracy comparison in Asians. Accuracies were measured based on matching in 4-digit level. We evaluated prediction accuracy in imputing HLA of Koreans (N = 413) using the merged panel of Chinese (N = 9,773) and HapMap EAS (N = 504) as reference. For HIBAG-prefit, we used the Asian prefit model. The error bars represent SD.

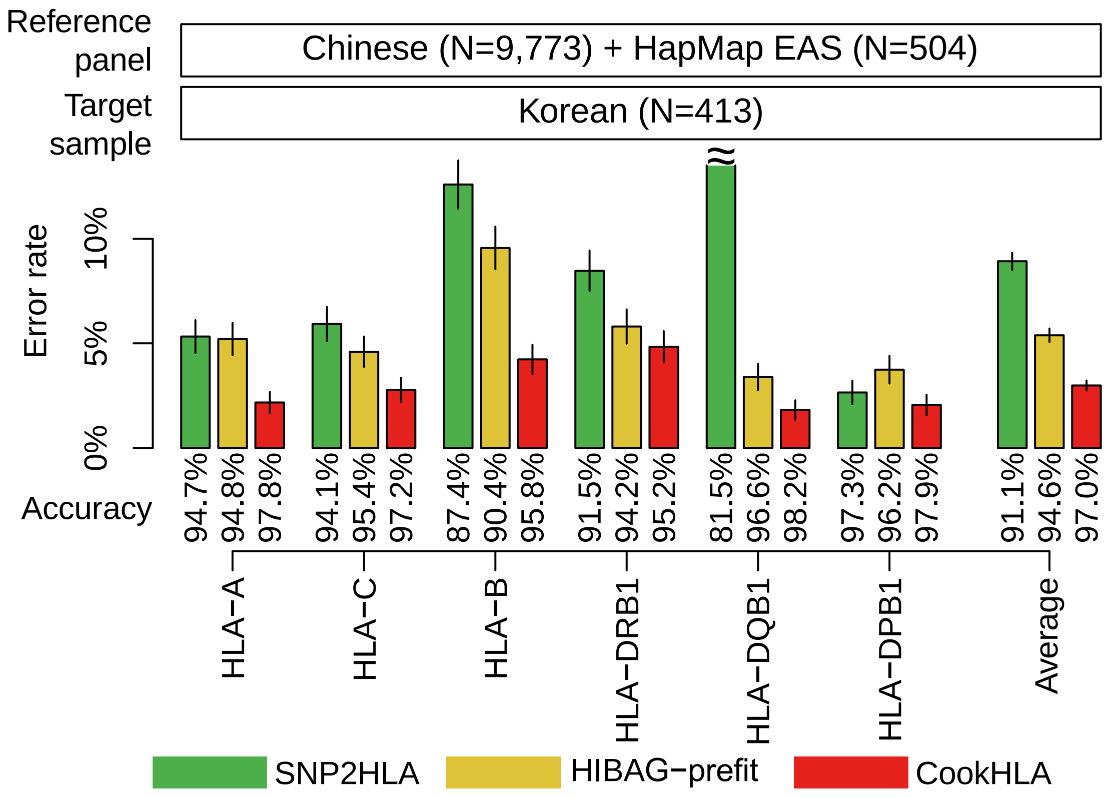

Supplementary Figure 2. Pairwise comparison across 10 different reference panels. We collected 10 reference panels of differing ethnicities and sizes. We then tested every possible pair by assigning one panel as reference and another as target, which comprised 88 tests after excluding overlapping sample pairs. **(a)** We compared the full version of CookHLA to CookHLA-vanilla (upgraded engine only) and **(b)** CookHLA-HapMap (upgraded engine, with HapMap genetic map) to CookHLA-vanilla. The dotted line indicates where the two methods' imputation accuracies are equal.

a.

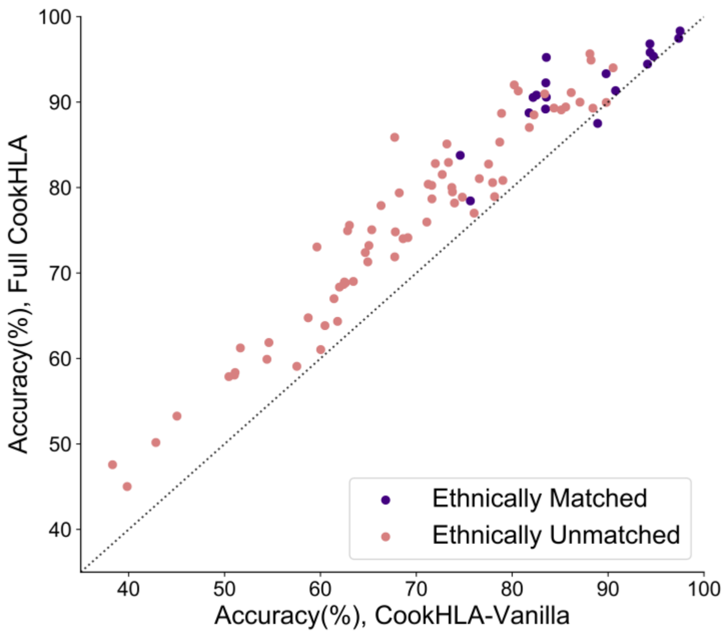

b.

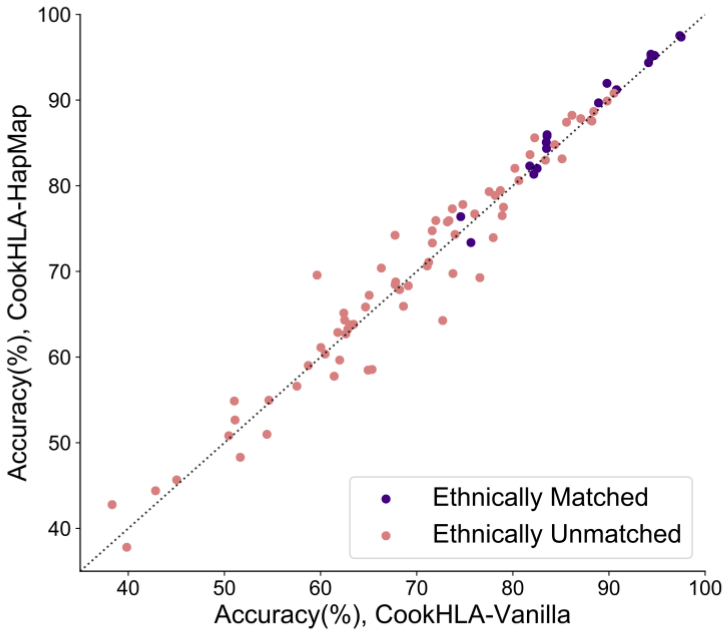

Supplementary Figure 3. Imputation accuracy of different versions of CookHLA in each allele frequency bin. (a) We used cross-validation by splitting the T1DGC panel into reference (N=1,000) and the target sample (N=4,225). **(b)** We used the 1000G EAS (N=504) as reference and the Korean data (N=413) as target. The accuracy refers to sensitivity.

a.

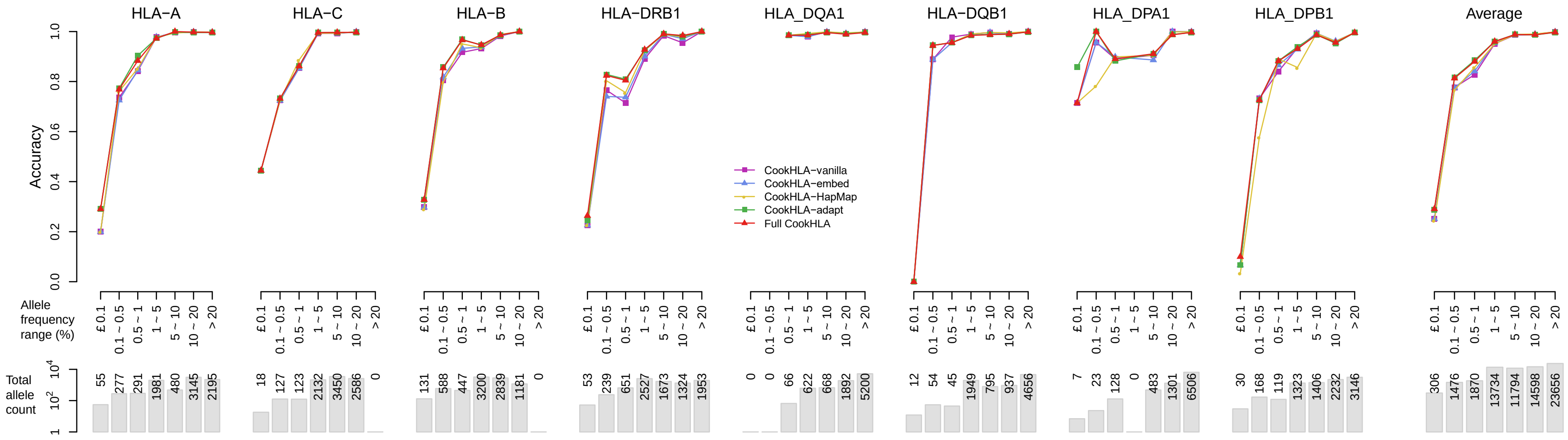

b.

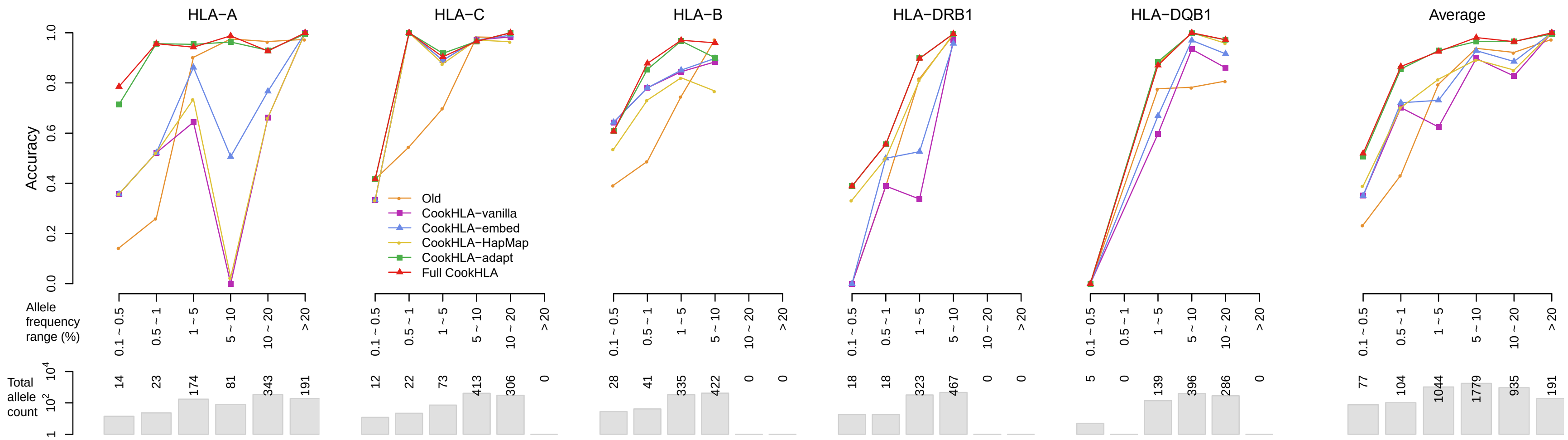

HLA−A
HLA−C
HLA−B
HLA−DRB1
HLA−DQB1
Average
Accuracy
Old
CookHLA−vanilla
CookHLA−embed
CookHLA−HapMap
CookHLA−adapt
Full CookHLA
Allele frequency range (%)
0.1 ~ 0.5
0.5 ~ 1
1 ~ 5
5 ~ 10
10 ~ 20
>20
Total allele count
HLA−A: 14 23 174 81 343 191
HLA−C: 12 22 73 413 306 0
HLA−B: 28 41 335 422 0 0
HLA−DRB1: 18 18 323 467 0 0
HLA−DQB1: 5 0 139 396 286 0
Average: 77 104 1044 1779 935 191

Supplementary Figure 4. Imputation accuracy (PPV, F1-score) in each allele frequency bin. We used cross-validation by splitting the T1DGC panel into reference (N=1,000) and the target sample (N=4,225). For HIBAG-prefit, we used the European prefit model. **(a)** PPV in each allele frequency bin. We omitted alleles with predicted allele counts of zero. **(b)** F1-score in each allele frequency bin. F1-score was defined as the harmonic mean of the sensitivity in Figure 4 and PPV. Only the alleles with non-zero sensitivity and PPV were included for calculation.

a.

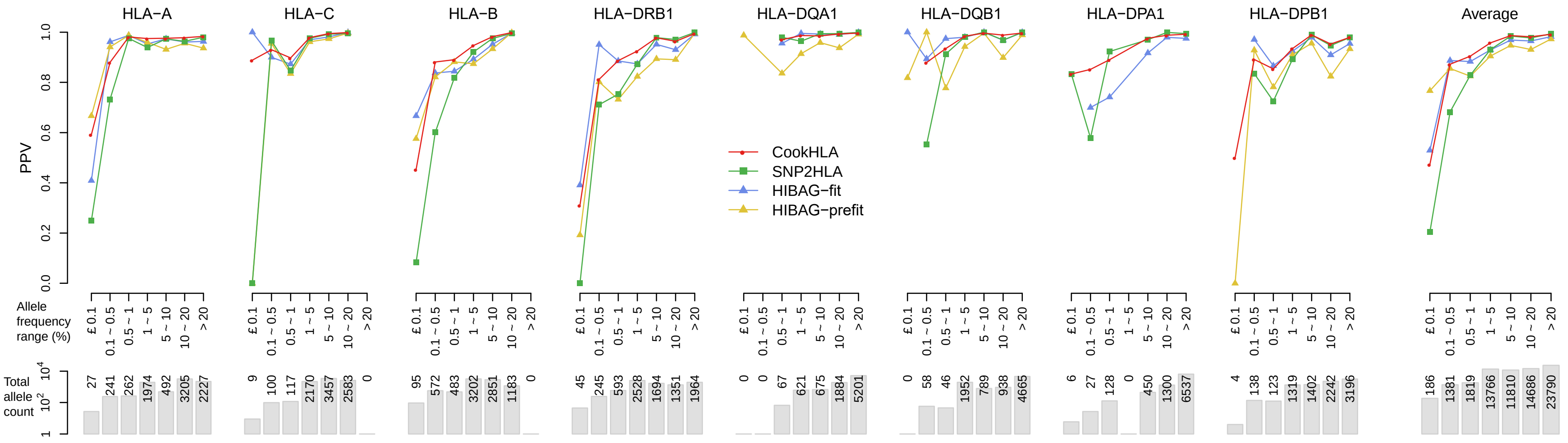

b.

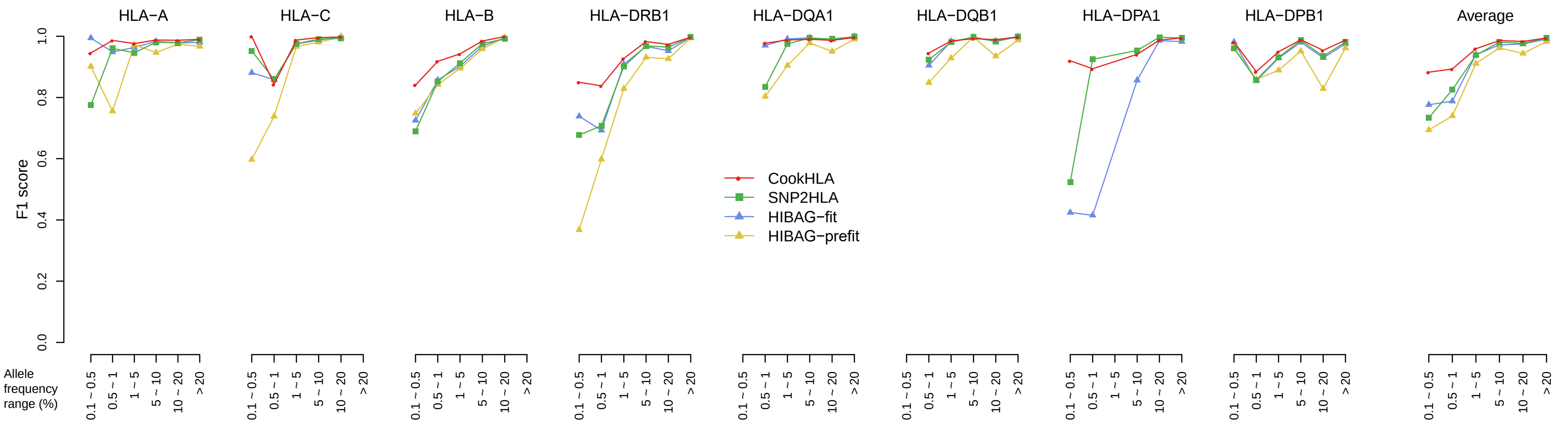
HLA−A
HLA−C
HLA−B
HLA−DRB1
HLA−DQA1
HLA−DQB1
HLA−DPA1
HLA−DPB1
Average
F1 score
0.0
0.2
0.4
0.6
0.8
1.0
CookHLA
SNP2HLA
HIBAG−fit
HIBAG−prefit
Allele frequency range (%)
0.1 ~ 0.5
0.5 ~ 1
1 ~ 5
5 ~ 10
10 ~ 20
> 20

Supplementary Figure 5. Imputation accuracy versus call rate. For each method, we changed the threshold to call genotypes based on the confidence score provided by the method. We used the T1DGC subset (N=1,000) as reference and the rest (N=4,225) as target, similar to **Figure 2C**. The imputation accuracy was averaged over all available HLA genes. (In SNP2HLA, *HLA-DPA1* was excluded because there was a phenomenon that all 4 digit markers show 0 posterior probabilities in this dataset.)

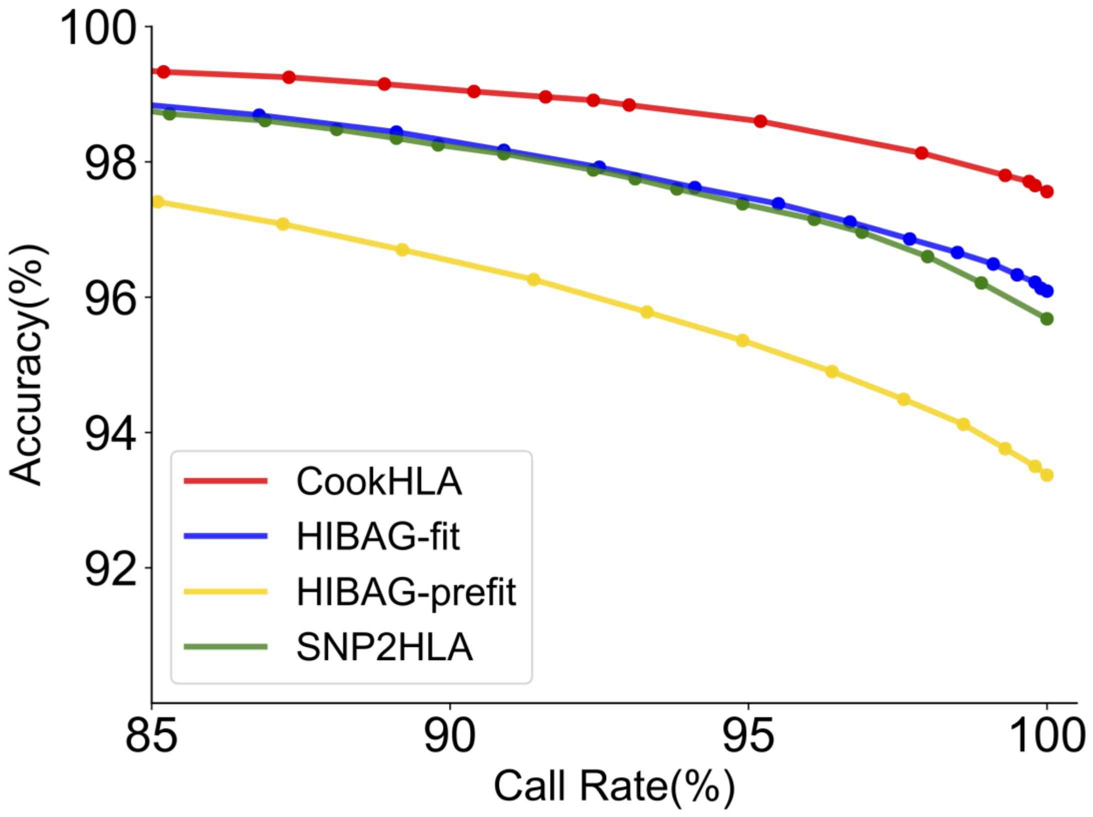

Supplement: Supplementary file 1 — Supplementary Information [file 41467_2021_21541_MOESM1_ESM.pdf]
