# Supplementary material for: Accurate imputation of human leukocyte antigens with CookHLA
Source: Nat Commun. 2021 Feb 24;12:1264. doi: 10.1038/s41467-021-21541-5 (PMC7904773; doi:10.1038/s41467-021-21541-5)
Supplement: Supplementary file 3 — Description of Additional Supplementary Files [file 41467_2021_21541_MOESM3_ESM.pdf]

Description of Additional Supplementary Files

File Name: Supplementary Data 1
Description: Imputation accuracy in each allele frequency bin. We used cross-validation by splitting the T1DGC panel into reference (N=1,000) and target sample (N=4,225). For HIBAG-prefit, we used the European prefit model. Accuracy refers to sensitivity.

File Name: Supplementary Data 2
Description: WTCCC association results of CD when CookHLA was used for imputation.

File Name: Supplementary Data 3
Description: WTCCC association results of RA when CookHLA was used for imputation.

File Name: Supplementary Data 4
Description: WTCCC association results of T1D when CookHLA was used for imputation.

File Name: Supplementary Data 5
Description: WTCCC association results of CD when SNP2HLA was used for imputation.

File Name: Supplementary Data 6
Description: WTCCC association results of RA when SNP2HLA was used for imputation.

File Name: Supplementary Data 7
Description: WTCCC association results of T1D when SNP2HLA was used for imputation.

File Name: Supplementary Data 8
Description: WTCCC association results of CD when HIBAG was used for imputation.

File Name: Supplementary Data 9
Description: WTCCC association results of RA when HIBAG was used for imputation.

File Name: Supplementary Data 10
Description: WTCCC association results of T1D when HIBAG was used for imputation.

File Name: Supplementary Data 11.
Description: Imputation accuracy and MACH subsample size. CookHLA internally uses MACH to estimate adaptive genetic map (AGM) with subsamples from the reference and target. We measured the imputation accuracy while varying the subsample size from 100/100 to 500/500. We benchmarked the three experiments presented in Figure 2. The results below show that increasing subsample size did not result in meaningful accuracy gain, despite largely increased computation time.
