# Supplementary material for: Accurate imputation of human leukocyte antigens with CookHLA
Source: Nat Commun. 2021 Feb 24;12:1264. doi: 10.1038/s41467-021-21541-5 (PMC7904773; doi:10.1038/s41467-021-21541-5)
Supplement: Supplementary file 15 — Reporting Summary [file 41467_2021_21541_MOESM15_ESM.pdf]

nature research

Corresponding author(s): Buhm Han

Last updated by author(s): Dec 19, 2020

Reporting Summary

Nature Research wishes to improve the reproducibility of the work that we publish. This form provides structure for consistency and transparency in reporting. For further information on Nature Research policies, see our Editorial Policies and the Editorial Policy Checklist.

Statistics

For all statistical analyses, confirm that the following items are present in the figure legend, table legend, main text, or Methods section.

n/a	Confirmed	
☐	☒	The exact sample size (n) for each experimental group/condition, given as a discrete number and unit of measurement
☐	☒	A statement on whether measurements were taken from distinct samples or whether the same sample was measured repeatedly
☐	☒	The statistical test(s) used AND whether they are one- or two-sided<br>*Only common tests should be described solely by name; describe more complex techniques in the Methods section.*
☐	☒	A description of all covariates tested
☐	☒	A description of any assumptions or corrections, such as tests of normality and adjustment for multiple comparisons
☐	☒	A full description of the statistical parameters including central tendency (e.g. means) or other basic estimates (e.g. regression coefficient) AND variation (e.g. standard deviation) or associated estimates of uncertainty (e.g. confidence intervals)
☐	☒	For null hypothesis testing, the test statistic (e.g. F, t, r) with confidence intervals, effect sizes, degrees of freedom and P value noted<br>*Give P values as exact values whenever suitable.*
☒	☐	For Bayesian analysis, information on the choice of priors and Markov chain Monte Carlo settings
☒	☐	For hierarchical and complex designs, identification of the appropriate level for tests and full reporting of outcomes
☐	☒	Estimates of effect sizes (e.g. Cohen's d, Pearson's r), indicating how they were calculated

Our web collection on statistics for biologists contains articles on many of the points above.

Software and code

Policy information about availability of computer code

Data collection	No software was used for the data collection of this study.
Data analysis	For imputation of Human Leukocyte Antigens, the following software packages were used: SNP2HLA v1.0.3 (http://software.broadinstitute.org/mpg/snp2hla/), HIBAG v1.24.0 (https://www.bioconductor.org/packages/release/bioc/html/HIBAG.html), and CookHLA v1.0 (https://github.com/WansonChoi/CookHLA; DOI:10.5281/zenodo.4294712). The internal procedure of CookHLA to obtain adaptive genetic map utilized MACH v1.0 (http://csg.sph.umich.edu/abecasis/MACH/tour/imputation.html). The HLA information of 1000 Genomes data was obtained from 1000 Genomes database (http://ftp.1000genomes.ebi.ac.uk/vol1/ftp/data_collections/HLA_types/), which states that HLA was processed with PolyPheMe (https://xegen.fr/polypheme). SNP2HLA internally uses beagle v3 (https://faculty.washington.edu/browning/beagle/b3.html), while CookHLA utilizes beagle v4 (https://faculty.washington.edu/browning/beagle/b4_0.html) and beagle v5 (https://faculty.washington.edu/browning/beagle/b5_0.html).

For manuscripts utilizing custom algorithms or software that are central to the research but not yet described in published literature, software must be made available to editors and reviewers. We strongly encourage code deposition in a community repository (e.g. GitHub). See the Nature Research guidelines for submitting code & software for further information.

Data

Policy information about availability of data

All manuscripts must include a data availability statement. This statement should provide the following information, where applicable:

- Accession codes, unique identifiers, or web links for publicly available datasets
- A list of figures that have associated raw data
- A description of any restrictions on data availability

All of the data sets analyzed in this manuscript are public and published in other papers.

We have referenced them in the manuscript and listed URLs at https://github.com/WansonChoi/CookHLA.

Field-specific reporting

Please select the one below that is the best fit for your research. If you are not sure, read the appropriate sections before making your selection.

☒ Life sciences ☐ Behavioural & social sciences ☐ Ecological, evolutionary & environmental sciences

For a reference copy of the document with all sections, see nature.com/documents/nr-reporting-summary-flat.pdf

Life sciences study design

All studies must disclose on these points even when the disclosure is negative.

Sample size	No specific size was determined.
Data exclusions	No data was excluded.
Replication	All replication attempts were successful at least twice because the software simulation provided the same results.
Randomization	Randomization was not used since there were no experimental groups.
Blinding	Blinding is not relevant to this study since no group allocation does not occur

Reporting for specific materials, systems and methods

We require information from authors about some types of materials, experimental systems and methods used in many studies. Here, indicate whether each material, system or method listed is relevant to your study. If you are not sure if a list item applies to your research, read the appropriate section before selecting a response.

Materials & experimental systems

n/a	Involved in the study	
☒	☐	Antibodies
☒	☐	Eukaryotic cell lines
☒	☐	Palaeontology and archaeology
☒	☐	Animals and other organisms
☒	☐	Human research participants
☒	☐	Clinical data
☒	☐	Dual use research of concern

Methods

n/a	Involved in the study	
☒	☐	ChIP-seq
☒	☐	Flow cytometry
☒	☐	MRI-based neuroimaging
